# Autochthonous Acid-Producing Bacteria from Catfish (*Clarias* sp.) with Antibacterial Activity against Selected Fish Pathogens: A Preliminary Study

**DOI:** 10.1155/2020/8526581

**Published:** 2020-02-21

**Authors:** Asep Awaludin Prihanto, Happy Nursyam, Yoga Dwi Jatmiko, Royani L. Hayati

**Affiliations:** ^1^Dept. Fishery Product Technology, Faculty of Fisheries and Marine Sciences, Brawijaya University. Jl. Veteran, Malang, East Java, Indonesia; ^2^Bio-Seafood Research Unit, Faculty of Fisheries and Marine Sciences, Brawijaya University. Jl. Veteran, Malang, East Java, Indonesia; ^3^Coastal and Marine Science Center, Brawijaya University. Jl. Veteran, 65145 East Java, Indonesia; ^4^Dept. Biology, Faculty of Mathematics and Natural Sciences, Brawijaya University. Jl. Veteran, Malang, East Java, Indonesia

## Abstract

In this study, the application of an autochthonous microorganism as probiotic on catfish (*Clarias* sp.) was scarcely reported. This study aimed to obtain probiotic candidates from the digestive tract (intestinal and gastric) of catfish. A total of nine isolates were successfully isolated from the catfish. Almost all bacterial colonies were morphologically round, had flat edges, were yellow, and produced clear zones as a sign of producing acid during culture. The analysis showed that the three isolates had the best activity in inhibiting fish pathogen isolates. Furthermore, molecular analysis revealed that those three isolates were *Bacillus velezensis* UB-C1, *Bacillus amyloliquifaciens* UB-C5, and *Bacillus cereus* UB-C8. Interestingly, those three bacteria were non-lactic acid bacteria.

## 1. Introduction

Catfish production in Indonesia is in a very declining state due to various constraints in cultivation, including the number of diseases and decrease in the quality of aquatic aquaculture environment and feed provided. According to Rachman et al. [1], the application of intensive cultivation systems resulted in the declining environment carrying capacity. The impact of this activity causes disruption to the balance of microorganism populations on the aquatic environment. This usually is one of the reasons for increase of pathogenic organisms including parasites, bacteria, and viruses which causes fish diseases. Various pathogenic bacteria such as *Vibrio* sp., *Aeromonas* sp., and *Pseudomonas* sp., will cause disease in cultivated fish; hence, it needs to be anticipated.

Several types of bacteria found in the digestive tract of animals have an important role in improving feed utilization, fish health, and environmental quality [2]. In addition, several bacteria in the digestive tract produce several types of enzymes which may play a vital role in host metabolism. The intestine and stomach are the main places for food and organs to be colonized by microbes that play a role or contribute to the process of food digestion and immune function. Therefore, by isolating the normal bacterial flora from these organs, it may support the finding of potential probiotic candidates. One alternative approach that has been successfully carried out for improving aquaculture productivity is introducing live bacteria that have already been known to have beneficial effects on the growth of aquatic animals, known as probiotics.

The origin bacteria which were found in the digestive tract have a mutual relationship with their host and use the host as their habitat. Many intestinal bacteria can synthesize vitamins, secrete enzymes, and help in digestion of nutrients, and the presence of native bacteria tends to suppress the growth of pathogenic bacteria, so they can protect the host and stimulate immune function [3–7]. This study aimed to determine the bacteria from the catfish (*Clarias* sp.) gastrointestinal tract that can be used for probiotics candidates against several pathogenic bacteria.

## 2. Materials and Methods

### 2.1. Fish Samples

Catfish (*Clarias* sp.) were purchased from a local fish farmer in Malang city, East Java, Indonesia. Fish samples were randomly collected from two ponds. Prior to the sampling, the ponds experienced an outbreak of diseases. Two survived catfishes were used as samples. Samples were immediately transported to a laboratory. Fish were aseptically dissected to take their intestine and stomach.

### 2.2. Isolation of Acid-Producing Bacteria

Isolation of digestive organs was carried out aseptically using surgical instruments. The stomach and intestines were removed from the fish. One gram samples were mildly crushed using mortal and put on 9 mL of saline solution. The diluted samples were plated onto de Man Rogosa-Sharpe (MRS) agar supplemented with 0.5% of CaCO_3_ [8]. Plates were incubated anaerobically in an anaerobic jar with an AnaeroGen^TM^ system for 48–96 hours at 37°C.

### 2.3. Identification of Bacteria

All isolates with a clear zone were transferred to the fresh MRS agar medium. Colonies showing different morphology such as color, form, and elevation were purely subcultured. Only isolates with the antagonism activity toward fish pathogenic bacteria were further identified by using the molecular method. Bacterial DNA was extracted using the method described by Murray (by following the company manual procedure). 16s rDNA were amplified by using universal primers 27f (5′- AGA GTT TGA TCC TGG CTC AG-3′) and 1492r (5′- GGT TAC CTT CTT ACG ACT T-3′) under PCR condition as follows: 35 cycles of 94°C for 5 mins, 94°C for 30 s, 55°C for 1 min, and 72°C for 90 s. Amplified nucleotides were purified using the QIA quick PCR purification kit (QIAGEN, GmbH, Germany). Prior to sequencing with Big Dye terminator sequencing on ABI PRISM 3100 DNA sequencer (Applied Biosystems), the amplified 16s rDNA were added with the sequenching primers, F1 (5′-GAGTTTGATCCTGGCT CAG-3′); F3 (5′-GTCCCGCAACGAGCGCAAC-3′); R1 (5′-GTATTACCGCGGCTGCTGCTG G-3′); R2 (5′-CATCGTTTACGGCGTGGAC-3′); R3 (5′-TTGCGCTCGTCGTTGCG GACT-3′); and R4 (5′-ACGGGCGGTGTGATACAAG-3′). Sequenced nucleotides were assembled using bioedit and aligned using ClustalX. The isolates identity was further checked using BLAST using the data on GenBank. The phylogenetic tree was performed followed the method of Dereeper et al. [9].

### 2.4. Antibacterial Assay

All isolates were screened for their antibacterial activity against clinical pathogen isolates. *Vibrio harveyi*, *V. alginolyticus*, *Edwardsiella tarda*, *Pseudomonas aeruginosa*, and *Aeromonas hydrophila* were obtained from Fish Disease Laboratory, Faculty of Fisheries and Marine Science, Brawijaya University. After 24 hours of incubation on the Luria Bertani broth medium, the medium was centrifuged on 2,500 rpm for 10 min at 4°C. Cell-free supernatants were tested for their antibacterial activity by using the disk-diffusion method [10]. The inhibition zones were observed after 24 hours of incubation.

## 3. Results and Discussion

Isolation of catfish-autochthonous bacteria was carried out to obtain potential candidates for fish probiotic. Digestive tract organs of catfish (stomach and intestines) were used as samples. According to Sugita et al. [11], fish intestine is the target organ for isolating aerobic and anaerobic heterophilic bacteria. The microflora from the fish digestive tract are capable of producing bioessential substances. For these reasons, the authochthonous bacteria are the important source for probiotic.

**Figure 1:**
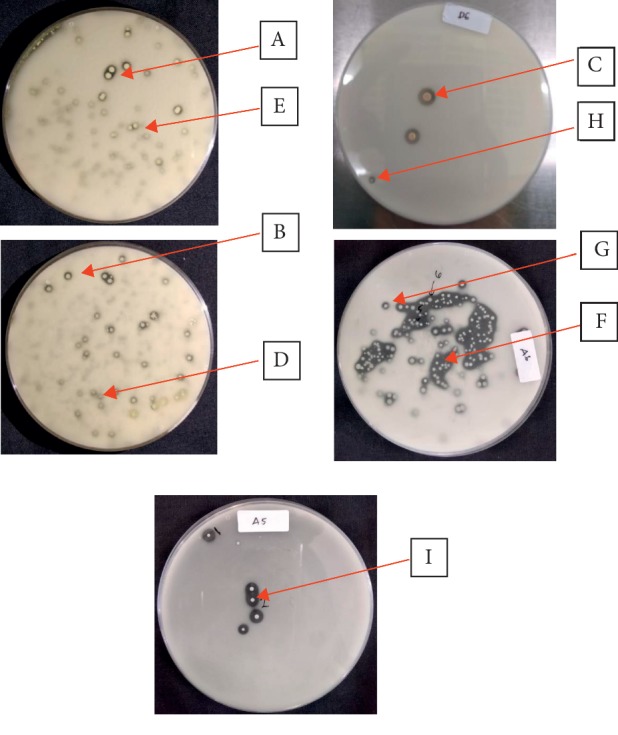
Selected colony morphology of catfish-autochthonous bacteria.

Nine isolates showed clear zones as an indication for producing acid in the medium (Figure 1). Addition of calcium carbonate (CaCO_3_) can be used for selecting lactic acid bacteria [12, 13]. The medium of MRSA is a common medium for isolating lactic acid bacteria. Among colonies, the morphological characteristics were as follows: a round colony form, flat edges, and white to creamy white color (Table 1).

Colony morphology and cell morphology of all bacterial isolates have almost the same character. All isolates were round shaped colonies. Most colonies were milky white and one was creamy white. Colony sizes ranged from 0.25 mm to 2.0 mm. The bacterial cells were rod-shaped and Gram-positive.

The pure isolates obtained were then tested for their antibacterial ability using *Vibrio harveyi*, *V. alginolyticus*, *Edwardsiella tarda*, *Pseudomonas aeruginosa*, and *Aeromonas hydrophila*. The antagonistic assay showed that only one bacterial isolate had broad-spectrum antibacterial activity (code UBL1) compared with other isolates (Table 2). Two bacterial isolates, namely UBL5 and UBL8 had an antibacterial activity against two indicator bacteria (*E. tarda* and *A. hydropjyla*).

Molecular identification was performed on three isolates which showed an antibacterial activity. The results of 16s rDNA analysis indicated that the isolates with codes of UBL1, UBL5, and UBL8 were *Bacillus velezensis*, *Bacillus amyloliquifaciens*, and *Bacillus cereus*, respectively. The sequence of the isolated bacteria, *Bacillus velezensis*, *Bacillus amyloliquifaciens*, and *Bacillus cereus*, was deposited into Genebank under the accession number of MN640841, MN640842, and MN640843, respectively.

The isolates were then compared with reference sequences species from NCBI. Phylogenetic analysis showed that all bacteria have high similarity with those from *Bacillus* species. Compared to the lactic acid-producing bacteria, such as *Lactobacillus* Casei and *L. paracasei*, those bacteria were quite different. *B. amyloliquifaciens* had a close relation with *B. velezensis.* Initially, *B. velezensis* was classified as *B. amyloliquefaciens,* yet during 2016-2017, they were reclassified [14].

This study was failed to specifically obtain lactic acid bacteria (*Lactobacillus* sp.). Inspite of the isolation using the MRSA medium, all three bacteria were identified as nonlactic acid bacteria. Targeted bacteria in this research are lactic acid bacteria. Hence, the MRSA medium was applied in all isolation procedures. The result indicated that non-lactic acid bacteria, *Bacillus* sp. were able to grow in the MRSA medium.

The clear zone around colonies indicated that the bacteria were able to produce acid substances. *Bacillus* sp. as non-lactic acid bacteria was competent in producing acids. Several *Bacillus* were able to produce notable amount of l-lactic acid bacteria either under aerobic or anaerobic condition [15–17]. This is the main reason for *Bacillus* sp. growth with a clear zone in MRS agar medium. The ability of *Bacillus* sp. to grow on MRS agar medium has been also confirmed by Poormontaseri et al. [18]. For this reason, it is noted that, on the basis of the medium composition, the MRS agar is not quite specific for the isolation of lactic acid bacteria from the gastrointestinal tract of catfish.

Gene cluster analysis on the *Bacillus* species revealed that PKS and NRPS gene cluster have existed only in *B. cereus.* Three gene clusters, Polyketide synthase (PKS), Nonribosomal peptide synthetase (NRPS) type I, and hybrid pathway, were found in *B. cereus* [19–21]. Furthermore, *B. velezensis* and *B. amyloliquefaciens* have an NRPS gene cluster. Thirteen secondary metabolite clusters were identified on *B. velezensis* CC09 and *B. amyloliquefaciens* [22]. Several productions of antimicrobial-volatile compounds, acetoin, and 2-butanone can also be produced by *B. velezensis* or *B. subtilis* [23].

*Bacillus* sp. is capable of spore-forming. Compared with the lactic acid bacteria, spore-forming bacteria have several advantages, such as a higher survival rate on the gastrointestinal tract and stability during processing and storage [24]. Therefore, acid-producing bacteria (*B. velezensis, B. cereus,* and *B. amyloliquefaciens*) are the best candidate for probiotic.

## 4. Conclusion

Nine bacterial isolates from catfish gastrointestinal tracts were isolated. Two isolates exhibited antibacterial activity on more than one tested bacteria. One isolate showed a broad spectrum antibacterial activity. We here reported that the *Bacillus velezensis* UBL1, *Bacillus amyloliquifaciens* UBL5, and *Bacillus cereus* UBL8 produce acids and they have an antibacterial activity. Acid and secondary metabolites are a plausible cause of antibacterial effect on those three bacteria.

## Figures and Tables

**Table 1:** Morphological characterization of purified isolates.

No	Colony observation							Gram
	Code	Colony size	Form	Color	Elevation	Edge	Surface	
1	C1	Small	Circular	Milky white	Flat	Entire	Smooth glistening	Positive
2	C2	Moderate	Circular	Milky white	Flat	Entire	Smooth glistening	Positive
3	C3	Small	Circular	Milky white	Flat	Entire	Smooth	Positive
4	C4	Small	Circular	Milky white	Flat	Entire	Smooth glistening	Positive
5	C5	Small	Circular	Milky white	Flat	Entire	Smooth glistening	Positive
6	C6	Moderate	Circular	Milky white	Flat	Entire	Smooth glistening	Positive
7	C7	Moderate	Circular	White	Flat	Entire	Smooth	Positive
8	C8	Small	Circular	Milky white	Flat	Entire	Smooth glistening	Positive
9	C9	Large	Circular	Creamy white	Flat	Entire	Smooth glistening	Positive

**Table 2:** Antibacterial activity of isolated bacteria from catfish (*Clarias* sp.).

No	Isolate code	Diameter of inhibition zone (mm)							
		*Vibrio harveyi*		*Vibrio alginolyticus*		*Edwardsiella tarda*		*Aeromonas hydrophila*	
		Original	Neutralized	Original	Neutralized	Original	Neutralized	Original	Neutralized
1	UBL1	19.1 ± 2.4	16.2 ± 4.9	18.4 ± 2.2	16.18 ± 1.98	6.3 ± 1.67	6.1 ± 1.1	17.8 ± 3.1	16.1 ± 2.2
2	UBL2		—		—	6.2 ± 0.3	6.2 ± 0.0		—
3	UBL3		—		—	6.4 ± 0.1	6.1 ± 0.0		—
4	UBL4		—		—	6.6 ± 1.1			—
5	UBL5		—		—	6.1 ± 0.1	6.1 ± 1.2		7.21 ± 3.6
6	UBL6		—		—	6.5 ± 0.0			—
7	UBL7		—		—	6.5 ± 0.1	6.1 ± 0.2		—
8	UBL8		—		—	6.1 ± 0.0	6.2 ± 0.2		6.1 ± 2.3
9	UBL9		—		—	6.8 ± 04	6.5 ± 0.2		—

Original: crude extract without pH adjustment. Neutralized: sample with neutralization (pH 7.0 ± 0.2).

## Data Availability

The data used to support the findings of this study are included within the article.
